# Preoperative Anaemia, Renal Function, and Operative Factors in Acute Kidney Injury and Mortality After Cardiac Surgery with a Prolonged ICU Stay: A Retrospective Cohort Study

**DOI:** 10.3390/jcm15124498

**Published:** 2026-06-10

**Authors:** Bedih Balkan, Engin İhsan Turan, Orçun Ünal, Lokman Yalçın

**Affiliations:** 1 Department of Anesthesiology and Reanimation, Intensive Care Unit, University of Health Sciences, Kanuni Sultan Süleyman Training and Research Hospital, İstanbul 34303, Türkiye; 2 Department of Anesthesiology and Reanimation, University of Health Sciences, Kanuni Sultan Süleyman Training and Research Hospital, İstanbul 34303, Türkiye; 3 Department of Cardiovascular Surgery, University of Health Sciences, Kanuni Sultan Süleyman Training and Research Hospital, İstanbul 34303, Türkiye; 4 Department of Cardiovascular Surgery, Tekirdağ Dr. İsmail Fehmi Cumalıoğlu City Hospital, Tekirdağ 59030, Türkiye; yalcinlokman@gmail.com

**Keywords:** cardiac surgery, acute kidney injury, preoperative anaemia, cardiopulmonary bypass, CKD-EPI eGFR, preoperative renal function, in-hospital mortality, ICU, multivariable logistic regression, KDIGO

## Abstract

Background: Acute kidney injury (AKI) is one of the more serious complications following cardiac surgery, consistently linked to prolonged mechanical ventilation and higher in-hospital mortality. This study examined whether preoperative anaemia and impaired renal function are associated with AKI and death in a high-risk cardiac-surgery cohort requiring extended postoperative ICU monitoring and how these associations behave after adjustment for procedure type and intraoperative variables. Methods: In this single-centre retrospective cohort study, we screened 950 patients admitted to a cardiothoracic ICU between January 2018 and January 2024. After standard exclusion criteria and an audit of operative records, 553 cardiac-surgery patients formed the principal analysis cohort. AKI was defined by KDIGO criteria using serial postoperative serum-creatinine measurements during the first 7 days. Multivariable logistic regression for AKI and in-hospital mortality was built sequentially: Model A (baseline only); Model B (+procedure type); and Model C (+intraoperative variables: aortic cross-clamp time, intraoperative RBC units, and intraoperative inotrope use). Calibration was assessed by the Hosmer–Lemeshow test. Total cardiopulmonary bypass duration was not separately captured in the institutional database and is disclosed as a limitation. Results: AKI occurred in 174 of 553 patients (31.5%), and in-hospital mortality was 16.6% (92/553). Patients with AKI were older (median 77 vs. 68 years, *p* < 0.001), with lower preoperative haemoglobin (11.4 vs. 12.3 g/dL, *p* < 0.001) and lower eGFR (38.1 vs. 63.7 mL/min/1.73 m^2^, *p* < 0.001). The aortic cross-clamp time was shorter in AKI patients (56 vs. 70 min, *p* = 0.043), a counterintuitive finding likely reflecting residual confounding by case-mix and procedure selection rather than a protective operative effect. In the fully adjusted multivariable model, the haemoglobin–AKI association attenuated and was no longer independently significant (OR 0.89 per 1 g/dL, 95% CI 0.73–1.08, *p* = 0.24), while intraoperative RBC transfusion emerged as an independent predictor (OR 1.12 per unit, *p* = 0.046). For mortality, AKI remained an independent predictor after full adjustment for procedure type and intraoperative variables (OR 7.14, 95% CI 1.45–35.13, *p* = 0.016), with cross-clamp time (OR 1.30 per 10 min, *p* = 0.010) and intraoperative RBC units (OR 1.48 per unit, *p* < 0.001) also independently associated. Both fully adjusted models showed acceptable calibration (Hosmer–Lemeshow *p* = 0.48 for AKI, *p* = 0.56 for mortality). Conclusions: In cardiac-surgery patients with a prolonged ICU stay, AKI is independently associated with in-hospital mortality even after adjustment for operative variables. The univariable association between preoperative haemoglobin and AKI is attenuated after adjustment for procedure type and intraoperative transfusion exposure, suggesting confounding or mediation by operative and case-mix factors rather than an independent direct association. The contribution of this analysis is aetiological/analytical rather than predictive (modest discrimination, AUROC 0.67 for AKI), and findings should be interpreted within the selected high-risk ICU ≥ 72 h population.

## 1. Introduction

Acute kidney injury following cardiac surgery affects a substantial proportion of patients, with reported rates ranging from 5% to 43% depending on the population studied, the surgical procedures included, and the diagnostic criteria applied [1,2,3]. Even modest postoperative increases in serum creatinine are associated with meaningfully worse short-term outcomes, including higher 30-day mortality and extended hospital stays [1,2,4].

The mechanisms underlying cardiac-surgery-associated AKI are multifactorial. Renal ischemia-reperfusion injury, systemic inflammation, haemolysis, and exposure to nephrotoxic agents during cardiopulmonary bypass each contribute to varying degrees [3,5,6,7]. Preoperative anaemia represents a separate pathway—through reduced oxygen-carrying capacity and increased transfusion exposure—that may amplify renal injury in the perioperative period [5,8,9,10,11], with perioperative blood-product exposure further compounding the risk [12,13,14]. Among preoperative factors, reduced kidney function is perhaps the most reliably identified risk marker: patients with pre-existing chronic kidney disease have less renal reserve and appear more vulnerable to perioperative haemodynamic insults, a pattern reflected in widely used risk-prediction scores [15,16].

Despite the substantial literature on this topic, several methodological issues recur across published studies: associations are frequently reported without multivariable adjustment for procedure type and intraoperative variables; eGFR and serum creatinine are sometimes entered simultaneously into regression models despite indexing the same construct; and missing-data patterns are often left unexamined. This study aimed to address these gaps in a large single-centre cohort, using CKD-EPI 2021 eGFR computed from routinely available data to minimize missingness and constructing parallel non-redundant regression models for both AKI and in-hospital mortality.

What this study adds: This study contributes to the cardiac-surgery AKI literature in two primarily analytical rather than descriptive ways. First, by applying sequential nested multivariable models we show how the apparent independent association between preoperative haemoglobin and AKI attenuates after adjustment for procedure type and intraoperative transfusion exposure—clarifying conditional independence rather than reporting a novel biological mechanism. Second, we explicitly characterise the AKI–mortality association in a selected high-risk ICU population (postoperative stay ≥ 72 h), with appropriate acknowledgment of the selection structure and its implications for causal interpretation.

## 2. Materials and Methods

### 2.1. Study Design and Setting

This is a retrospective observational cohort study conducted at a single tertiary academic cardiac-surgery centre. Patients who underwent cardiac surgery between January 2018 and January 2024 were eligible for inclusion. The study was approved by the institutional ethics committee (IRB No: 2024.07.155) and conducted in accordance with the Declaration of Helsinki. Given the retrospective design, the requirement for individual informed consent was waived. All data were de-identified prior to analysis. Reporting follows the STROBE guidelines for observational studies.

### 2.2. Participants

**Figure 1:**
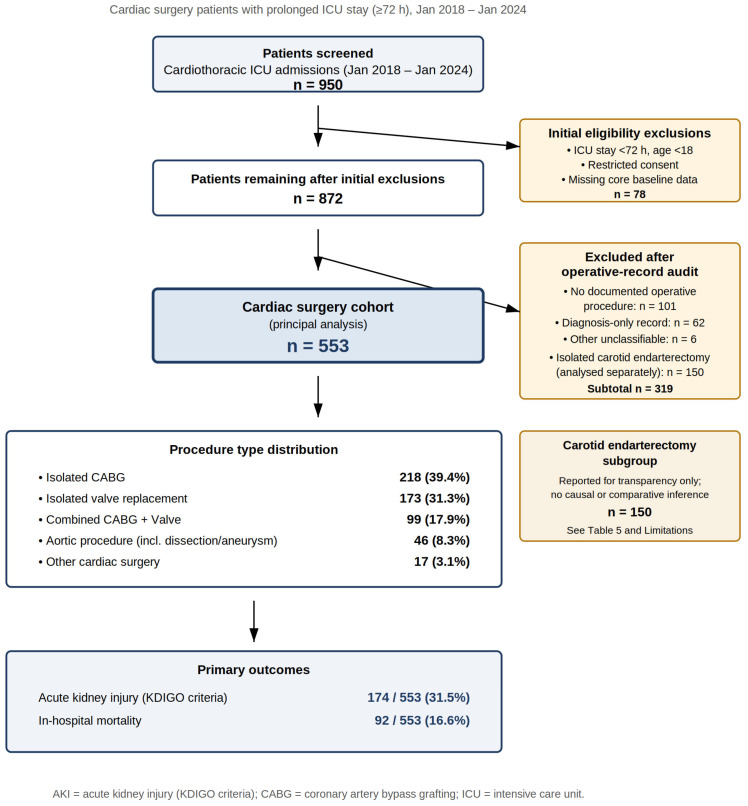
Patient selection flow diagram. Of the 950 patients screened, 553 cardiac-surgery patients formed the principal analysis cohort after exclusion of 78 by initial criteria and 319 by operative-record audit (101 no documented operative procedure, 62 diagnosis-only, 6 unclassifiable, and 150 isolated carotid endarterectomy, reported separately (see Limitations)). Outcomes in the final cohort: AKI, 174 (31.5%); in-hospital mortality, 92 (16.6%).

The patient selection flow diagram is shown in Figure 1. The carotid endarterectomy subgroup is reported for transparency only (see Limitations). The directed acyclic graph (DAG; constructed using DAGitty [17]) representing the assumed causal structure underlying the analysis is provided as Supplementary Figure S1.

Exclusion criteria were: age under 18 years; absent baseline renal function tests; history of nephrectomy or renal transplantation; preoperative mechanical circulatory support; uneventful early ICU discharge within 24 h of surgery; ASA class VI; and restricted consent for observational data use.

Inclusion criterion of ICU stay ≥72 h: Selection considerations: The ≥72 h ICU stay criterion was applied to identify patients who required sustained postoperative monitoring and for whom complete perioperative laboratory datasets were available for KDIGO AKI staging. We acknowledge that this criterion produces a high-risk subgroup with AKI incidence and mortality rates substantially higher than those reported in unselected cardiac-surgery cohorts. We further acknowledge that the criterion may act as a selection variable/collider influenced both by preoperative risk factors and by postoperative complications (including AKI itself), as illustrated in the directed acyclic graph (Supplementary Figure S1) [17]. Findings should therefore be interpreted within this high-risk subgroup; estimates may carry residual collider-induced bias and should not be generalised to unselected cardiac surgery populations.

Procedure type composition: In the final cardiac-surgery cohort: Isolated CABG, 218 (39.4%); Isolated valve replacement, 173 (31.3%); Combined CABG + Valve, 99 (17.9%); Aortic procedure, 46 (8.3%, including 5 aortic dissections and 1 ruptured aneurysm); and Other cardiac surgery, 17 (3.1%). ASA classification was recorded for 396/553 (71.6%) patients with ASA ≥IV in 19.3% of patients. An explicit elective vs. emergency flag was not systematically coded; unambiguous emergency presentations identifiable from operative free text are listed above.

### 2.3. eGFR Calculation

Hospital-recorded GFR values were missing for a substantial proportion of patients, predominantly an artefact of progressive electronic-record adoption (paper records prior to mid-2020) rather than systematic non-recording in sicker patients. To minimize missingness, eGFR was computed using the CKD-EPI 2021 race-free equation [18] from serum creatinine, age, and sex; this yielded valid values for ≥99% of patients. Throughout the manuscript, eGFR refers to this computed CKD-EPI 2021 value unless otherwise specified.

### 2.4. Definitions

AKI was defined according to KDIGO criteria [19] using serial postoperative serum-creatinine measurements during the first 7 days after surgery. The earliest creatinine increment meeting Stage 1 criteria (≥0.3 mg/dL within 48 h or ≥1.5–1.9× baseline within 7 days) was used to assign AKI status. Stage 2 (2.0–2.9× baseline) and Stage 3 (≥3.0× baseline, ≥4.0 mg/dL, or initiation of renal-replacement therapy) were assigned by the highest creatinine recorded during the postoperative 7-day window. AKI staging was driven principally by serum-creatinine criteria; hourly urine-output records were available but not consistently reliable across the 6-year study period (with progressive transition between paper and electronic ICU charting in 2020). The urine-output criterion was applied only in the relatively small fraction of patients with documented sustained oliguria. AKI events occurring after hospital discharge were not captured. Preoperative anaemia was defined per WHO criteria (Hb <13 g/dL in males; <12 g/dL in females). The primary outcome was AKI by KDIGO criteria; the secondary outcome was in-hospital mortality. Procedure type was extracted from operative free text and classified as Isolated CABG, Isolated valve replacement, Combined CABG + Valve, Aortic procedure, or Other cardiac surgery. Intraoperative variables included aortic cross-clamp time, intraoperative red-cell transfusion (units), intraoperative fresh-frozen plasma (units), and intraoperative inotrope/vasopressor use (binary, with a sub-breakdown by agent in Table 1). Total cardiopulmonary bypass duration was not separately captured in the institutional database during the study period (paper perfusion records prior to mid-2020); aortic cross-clamp time is the available proxy, and this is disclosed as a limitation.

On-pump vs off-pump status: Off-pump coronary artery bypass grafting (OPCAB) was not recorded as a distinct technique in the institutional database during the study period (no OPCAB, off-pump, or beating-heart entries appear in the operative free text), and based on operative record review and institutional practice during the study period no off-pump CABG cases were identified; all CABG procedures were performed using standard on-pump cardiopulmonary bypass with aortic cross-clamping. This is consistent with the institutional practice during the study period, in which off-pump CABG was not a routine technique. Aortic cross-clamp time is therefore available for the majority of procedures (482/553, 87%); the 13% of cases without a documented cross-clamp time reflect paper-based perfusion records (predominantly pre-mid-2020) that were not systematically digitised, rather than off-pump surgery.

### 2.5. Statistical Analysis

Continuous variables were summarised as medians [IQR] and compared between AKI and non-AKI groups with the Mann–Whitney U test; categorical variables are presented as n (%) and were compared with the Chi-square test or Fisher’s exact test (when expected cell counts < 5). Statistical significance was set at two-sided *p* < 0.05. Throughout the manuscript, *p*-values are reported as *p* = 0.XXX (3 decimal places) for values ≥ 0.001 and as *p* < 0.001 for smaller values.

Multivariable logistic regression for AKI and for in-hospital mortality was built sequentially in three nested models: **Model A** (baseline: age, sex, hypertension, diabetes, COPD, preoperative haemoglobin, and preoperative eGFR); **Model B** (Model A + procedure type, with Isolated CABG as reference); **Model C** (Model B + intraoperative aortic cross-clamp time per 10 min, intraoperative RBC units per unit, and intraoperative inotrope use as a binary). Effect sizes are reported in clinically interpretable units (per 1 g/dL haemoglobin, per 10 mL/min/1.73 m^2^ eGFR, and per 10 min cross-clamp). Sample sizes for each model are reported transparently because variable-specific missingness produced different complete-case sub-samples (Models A, B: n = 392; Model C: n = 235). The ‘Other cardiac surgery’ procedure category (n = 17, 1 AKI event) was excluded from regression modelling to avoid quasi-separation but is reported descriptively in Table 1. Non-linearity was tested formally using a likelihood-ratio test comparing linear and quadratic specifications for each continuous predictor (haemoglobin, eGFR, and creatinine). Model discrimination was assessed by the area under the ROC curve (AUROC); model calibration by the Hosmer–Lemeshow goodness-of-fit test (deciles of predicted risk). Decision-curve analysis was considered but not performed: the manuscript is framed as aetiological/analytical rather than as a clinical-prediction-rule derivation. As a sensitivity analysis, the most-complete-data subset (age, sex, comorbidities, and haemoglobin) was modelled on the full 553-patient cohort and yielded conclusions consistent with the principal model. Multiple imputation was considered but not pursued because the missingness pattern is dominated by a temporal/administrative mechanism (paper records pre-2020) rather than a missing-at-random conditional on observed variables. Variables with missingness >50% (sodium, potassium, HbA1c, and lactate) were excluded a priori from multivariable modelling and are reported descriptively only. All analyses were performed with Python 3.10 (statsmodels 0.14, scikit-learn 1.3, scipy 1.11) and SPSS v22.0. An AI-based large language model (Claude, Anthropic) was used for language and grammar editing of the manuscript text; no AI tools were used in the design, statistical analysis, data interpretation, or conclusions of the study.

## 3. Results

### 3.1. Demographic and Clinical Characteristics

Of the 553 cardiac-surgery patients in the principal analysis cohort, 174 (31.5%) developed postoperative AKI. Baseline characteristics, intraoperative variables, and postoperative outcomes are presented in Table 1. Median age was 69 years (IQR 60–77), and 61.1% were male. Comorbidities were common: hypertension, 52.6%; diabetes, 31.8%; and COPD, 12.3%. Patients with AKI were significantly older (median, 77 vs. 68 years, *p* < 0.001), with lower preoperative haemoglobin (11.4 vs. 12.3 g/dL, *p* < 0.001) and lower preoperative eGFR (38.1 vs. 63.7 mL/min/1.73 m^2^, *p* < 0.001).

### 3.2. Laboratory Findings and Clinical Outcomes

Table 2 presents additional preoperative and postoperative laboratory values and clinical outcomes. Preoperative CKD-EPI eGFR (computed) was substantially lower in AKI patients (median, 38.1 vs. 63.7 mL/min/1.73 m^2^, *p* < 0.001), and BUN was higher (25 vs. 21 mg/dL, *p* = 0.006).

Preoperative serum creatinine, presented as the median [IQR] consistent with its non-normal distribution, did not show a clinically meaningful difference between AKI and non-AKI groups (1.1 vs. 1.0 mg/dL).This explains why median-creatinine comparisons appear similar between groups despite their multivariable contribution. CKD-EPI eGFR—which itself includes a non-linear age-creatinine transformation—is therefore preferred as the principal renal-function variable.

AKI patients required markedly longer mechanical ventilation (median, 72 h, IQR, 27–82, vs. 17 h, IQR 10–22; *p* < 0.001) and substantially worse postoperative renal function (postoperative creatinine, 1.9 vs. 1.2 mg/dL; postoperative eGFR, 27.0 vs. 62.0 mL/min/1.73 m^2^, both *p* < 0.001). In-hospital mortality was 21.8% in AKI patients vs. 14.2% in non-AKI patients (*p* = 0.035). An important—and at first counterintuitive—observation is that the aortic cross-clamp time was shorter in AKI patients (median, 56 vs. 70 min, *p* = 0.043). This finding likely reflects residual confounding by case-mix and procedure-selection effects rather than a protective operative effect: AKI patients were older (median 77 vs. 68 years), had a lower baseline eGFR (38.1 vs. 63.7 mL/min/1.73 m^2^), and underwent shorter, less complex procedures more frequently than non-AKI patients (Isolated CABG, 52.9% vs. 33.2%, *p* < 0.001; Isolated valve 23.0% vs. 35.1%; *p* = 0.006). The pattern indicates that case-mix differences rather than procedural duration dominated AKI risk in this cohort.

### 3.3. Missing Data

Variable-specific missingness in the cardiac-surgery cohort was predominantly an artefact of progressive electronic-record adoption (paper records prior to mid-2020) rather than systematic non-recording in sicker patients. Major patterns are as follows: hospital-recorded GFR, 27% missing (resolved by computing CKD-EPI eGFR for ≥99%); aortic cross-clamp time, 13% missing; intraoperative RBC units, 31% missing; LVEF, 27% missing; ASA classification, 28% missing; postoperative drainage, 60% missing; and sodium/potassium/HbA1c/lactate, 75–80% missing (excluded a priori from multivariable models). Detailed reasons are tabulated in the Supplementary Methods. The principal logistic regression models were fitted on complete cases for each model specification (Models A and B: n = 392; Model C: n = 235); sample sizes are reported transparently in Table 3 and Table 4.

### 3.4. Multivariable Logistic Regression—AKI

Sequential multivariable logistic regression models for AKI are presented in Table 3. In the univariable analysis, preoperative haemoglobin was strongly associated with AKI (OR 0.86 per 1 g/dL increase, 95% CI 0.79–0.94, *p* < 0.001; equivalent to OR 1.16 per 1 g/dL *decrease*), as was eGFR (OR 0.99 per mL/min, *p* = 0.001; equivalent to OR 1.15 per 10 mL/min decrease) and intraoperative RBC units (OR 1.09 per unit, *p* = 0.015). In Model A (baseline only, n = 392), eGFR remained an independent predictor (OR 0.99, *p* = 0.023), but the haemoglobin association attenuated to non-significance (OR 0.91, *p* = 0.124). Adjusting for procedure type (Model B) did not appreciably change these estimates. In the fully adjusted Model C (adding cross-clamp time, intraoperative RBC units, and intraoperative inotrope; n = 235), only intraoperative RBC transfusion remained independently associated with AKI (OR 1.12 per unit, *p* = 0.046). Intraoperative inotrope use showed an inverse association in the complete-case model (OR 0.32, 95% CI 0.13–0.81, *p* = 0.016); given the counterintuitive direction and the possibility of documentation, selection, and complete-case effects, this finding was not interpreted as biologically protective. Haemoglobin (OR 0.89, *p* = 0.24) and eGFR (OR 0.99, *p* = 0.16) lost statistical significance after full adjustment. Discrimination and calibration results were as follows: AUROC for the fully adjusted AKI Model C was 0.672—which is modest and consistent with a multifactorial process. The Hosmer–Lemeshow goodness-of-fit test was non-significant (chi^2^ = 7.56, df = 8, *p* = 0.478), indicating acceptable calibration.

### 3.5. Multivariable Logistic Regression—In-Hospital Mortality

Sequential multivariable logistic regression models for in-hospital mortality are presented in Table 4. AKI was a strong predictor of mortality even in the crude analysis (OR 1.74, 95% CI 1.09–2.79, *p* = 0.021). After adjustment for age, sex, comorbidities, haemoglobin and eGFR (Model A), the adjusted OR was 1.98 (*p* = 0.041). In the fully adjusted Model C (adding procedure type, cross-clamp time and intraoperative RBC units; n = 235), the AKI–mortality association became substantially stronger: AKI OR 7.14, 95% CI 1.45–35.13, *p* = 0.016—a result robust to the inclusion of operative covariates and procedure type. Aortic cross-clamp time (OR 1.30 per 10 min, *p* = 0.010) and intraoperative RBC units (OR 1.48 per unit, *p* < 0.001) also emerged as independent predictors. The mortality model showed strong discrimination (AUROC 0.925) and acceptable calibration (Hosmer–Lemeshow, *p* = 0.559). The model fit improved markedly with the addition of operative variables (Pseudo R^2^ 0.072 → 0.452).

Non-linearity testing for continuous predictors is presented in Table 5. Likelihood-ratio tests comparing linear and quadratic specifications confirmed that haemoglobin and eGFR are well-described as linear predictors for AKI (LR *p* = 0.84 and *p* = 0.55 respectively). Serum creatinine, however, showed clear non-linearity (LR chi^2^ = 13.13, *p* < 0.001), consistent with a threshold effect (Table 6): the AKI rate is modest across the lower creatinine range and rises at >2.0 mg/dL. This supports the use of CKD-EPI eGFR—which itself includes a non-linear age-creatinine transformation—as the principal renal-function variable.

Effect sizes in clinically interpretable units and the categorical creatinine analysis are summarised in Table 6. The Hb–AKI association corresponds to OR 1.16 per 1 g/dL decrease (univariable, 95% CI 1.07–1.26); the eGFR–AKI association to OR 1.15 per 10 mL/min decrease (univariable, 95% CI 1.06–1.24). The categorical creatinine analysis confirms a threshold pattern with the highest AKI rate (36.6%) in patients with creatinine >2.0 mg/dL.

Descriptive characteristics of the cardiac-surgery cohort and the carotid endarterectomy subgroup are presented in Table 7.

## 4. Discussion

In this single-centre retrospective cohort of 553 cardiac-surgery patients with prolonged ICU stay, AKI was common (31.5%) and was a strong independent predictor of in-hospital mortality even after adjustment for procedure type and intraoperative variables (fully adjusted OR 7.14) [4,20]. The univariable association between preoperative haemoglobin and AKI—clear and statistically significant in the unadjusted analysis—attenuated substantially after full multivariable adjustment, and the conditional independent contribution of haemoglobin to AKI risk in this population appears to be largely mediated by perioperative transfusion exposure and baseline frailty. Our findings can be quantitatively benchmarked against prior cardiac-surgery AKI literature. The reported AKI incidence (31.5%) falls within the 5–43% range reported by Hu et al. and others [1] and at the higher end is consistent with our selection of patients with prolonged (≥72 h) ICU stay. The univariable haemoglobin–AKI OR (0.86 per 1 g/dL) is consistent in magnitude with Karkouti et al. (OR 0.85–0.92 in a multi-centre cardiac-surgery cohort) [8]. The fully adjusted AKI–mortality OR of 7.14 falls at the higher end of the 1.5–8 range reported in published cohorts [2]; the higher value plausibly reflects the prolonged-ICU-stay selection.

Procedure-type pattern and the counterintuitive cross-clamp finding: Two findings in our procedure-type analysis warrant comment. First, the aortic cross-clamp time was shorter in AKI patients (median 56 vs. 70 min), which appears to contradict the literature linking prolonged cross-clamp times with renal injury [6,21,22]. The most likely explanation is selection by case-mix: AKI patients were older, had a lower baseline eGFR, and were disproportionately offered shorter, less complex procedures (more often isolated CABG than complex valvular or aortic surgery). In the fully adjusted multivariable model for in-hospital mortality, cross-clamp time was a significant independent predictor (OR 1.30 per 10 min, *p* = 0.010), recovering the expected biological direction once procedural complexity and patient-mix are accounted for. Second, the lower observed AKI rate in patients undergoing isolated valve replacement (23.0% vs. 42.2% in isolated CABG) appears counterintuitive—valve patients are often considered to have more complex perioperative physiology. However, our valve cohort was younger, less frequently anaemic, and had a higher baseline eGFR than the isolated CABG cohort; this case-mix difference is the most plausible explanation for the lower observed AKI incidence rather than a procedure-specific protective effect of valvular surgery. Both findings emphasise the importance of stratifying by procedure type and adjusting for baseline differences when interpreting operative-variable–outcome associations in cohort studies.

Preoperative anaemia and AKI: Lower preoperative haemoglobin showed a strong univariable association with AKI (OR 0.86 per 1 g/dL, *p* < 0.001), but this association attenuated and lost statistical significance after full multivariable adjustment (OR 0.89, *p* = 0.24). Intraoperative red-cell transfusion emerged as an independent predictor (OR 1.12 per unit, *p* = 0.046), suggesting that the univariable haemoglobin signal is at least partly mediated by perioperative transfusion exposure [10,11], with potential additional confounding or mediation by operative and case-mix factors that we could not measure directly. This pattern is consistent with the hypothesis advanced by Karkouti and colleagues that the apparent association between preoperative anaemia and AKI is, at least in part, mediated by the cumulative effect of anaemia plus perioperative transfusion exposure on renal oxygen delivery and inflammatory injury [8,10].

Renal dysfunction and AKI/mortality: Preoperative eGFR was univariably associated with AKI (OR 1.15 per 10 mL/min decrease, *p* = 0.001) but lost statistical significance in the fully adjusted Model C (*p* = 0.16). Serum creatinine showed a non-linear (threshold) relationship with AKI: The AKI rate is approximately stable across the lower creatinine range but rises in the >2.0 mg/dL category (Table 6). This explains why median-creatinine comparisons appear similar between AKI and non-AKI groups despite the multivariable contribution of renal function—the right tail of the distribution carries disproportionate AKI risk. CKD-EPI eGFR, which itself includes a non-linear age-creatinine transformation, captures renal function more robustly than raw creatinine in linear regression [2,18].

AKI and in-hospital mortality: AKI was independently associated with substantially increased risk of in-hospital mortality across all model specifications: crude OR 1.74 → adjusted OR 1.98 → fully adjusted OR 7.14 (95% CI 1.45–35.13, *p* = 0.016). The robustness of this finding to the inclusion of operative covariates is, in our view, the most clinically meaningful result of the analysis [4,20]. Cross-clamp time (OR 1.30 per 10 min, *p* = 0.010) and intraoperative RBC units (OR 1.48 per unit, *p* < 0.001) also emerged as independent mortality predictors, consistent with prior reports linking procedural complexity and perioperative transfusion exposure with worse outcomes [5,12,21].

Selection of the high-risk ICU subgroup and its implications: The ≥72 h ICU stay criterion produces a high-risk subgroup with an AKI incidence (31.5%) and in-hospital mortality (16.6%) that are substantially higher than those reported in unselected cardiac-surgery cohorts (typically 5–20% AKI and <5% mortality). Critically, ICU stay ≥72 h is plausibly influenced both by preoperative risk factors and by postoperative complications, including AKI itself. Conditioning on this selection variable opens a non-causal path between preoperative covariates and AKI through the selection step—a potential collider effect illustrated in the directed acyclic graph (Supplementary Figure S1). Within-sample associations may therefore reflect, in part, collider-induced statistical dependence rather than purely causal effects. We frame the principal results as conditional associations within the selected sample and emphasise that estimates should not be generalised to unselected cardiac-surgery populations. Future prospective work in unselected cardiac-surgery cohorts would clarify the magnitude of any collider-induced bias.

eGFR computation and missing data: Computing CKD-EPI eGFR from creatinine, age, and sex resolved the substantial missingness in hospital-recorded eGFR values (≥99% complete after computation) and used the most contemporary race-free 2021 equation [18]. Variable-specific missingness in operative variables (cross-clamp time 13%, intraoperative RBC units 31%) reflects progressive electronic-record adoption (paper records pre-2020) rather than a missing-at-random conditional on observed variables. We addressed this analytically by reporting complete-case sample sizes for each model specification and conducted sensitivity analysis on the most-complete-data subset; conclusions were consistent. Multiple imputation was considered but not pursued because the missingness mechanism is dominated by a temporal/administrative source.

Limitations: This study has several important limitations. First, the single-centre retrospective design limits external generalisability. Second, total cardiopulmonary bypass duration was not separately captured in the institutional database (paper perfusion records prior to mid-2020); aortic cross-clamp time is the available proxy. Third, an explicit elective vs. emergency flag was not systematically recorded; ASA classification (recorded for 71%) and explicit identification of aortic dissection cases (n = 5) provide indirect indicators only. Fourth, preoperative pharmacological therapy (beta-blockers, ACE/ARB management, antiplatelet management, and iron/erythropoietin supplementation) was not captured in a structured form. Preoperative iron/erythropoietin supplementation was not part of the standard institutional protocol. Fifth, detailed surgical complexity scoring (EuroSCORE II, STS) was not available in standardised form. Sixth, AKI staging was driven principally by serum-creatinine criteria; hourly urine-output records were available but not consistently reliable across the 6-year study period, and AKI events occurring after hospital discharge were not captured. Seventh, the ≥72 h ICU criterion produces a selected high-risk subgroup; conditioning on this selection variable may induce collider bias. Eighth, the high AUROC of the fully adjusted mortality model (0.925) should be interpreted cautiously because it was estimated in a smaller complete-case subset (n = 235) and may be subject to optimism and selection effects; the model was not internally validated for clinical prediction. Decision-curve analysis was not performed; we frame the contribution as aetiological/analytical rather than predictive. Ninth, the ‘Other cardiac surgery’ category was small (n = 17, 1 AKI event) and was excluded from regression modelling to avoid quasi-separation. Tenth, baseline frailty was not measured directly; references to case-mix or frailty in the Discussion are interpretive and reflect indirect indicators (age, eGFR, and procedure type) rather than a validated frailty index [23]. Eleventh, the carotid endarterectomy subgroup (Table 7) is reported for transparency only; the two groups differ substantially in clinical complexity, baseline risk, and operative exposure (cardiopulmonary bypass vs. no bypass) and are therefore not directly comparable. No causal inference is drawn from this subgroup, and a formal comparative analysis would warrant a dedicated study design. Findings should be interpreted within the high-risk ICU population studied and emphasise the value of explicit consideration of intraoperative variables when interpreting preoperative AKI risk markers in clinical and research settings.

## 5. Conclusions

In cardiac-surgery patients with a prolonged postoperative ICU stay, AKI is a frequent (31.5%) complication and is independently associated with in-hospital mortality (fully adjusted OR 7.14) even after controlling for procedure type and intraoperative variables. The univariable association between preoperative haemoglobin and AKI is attenuated after adjustment for procedure type and intraoperative transfusion exposure, suggesting confounding or mediation by operative and case-mix factors rather than an independent direct association. The contribution of this analysis is aetiological/analytical rather than predictive (modest discrimination, AUROC 0.67 for AKI). Findings should be interpreted within the selected high-risk ICU ≥ 72 h population and emphasise the value of explicit consideration of intraoperative variables when interpreting preoperative AKI risk markers in clinical and research settings. Routine consideration of intraoperative blood-management strategies (transfusion thresholds, antifibrinolytics, and intraoperative cell salvage) may have a higher yield than reliance on preoperative haemoglobin alone as a discriminator of AKI risk.

## Figures and Tables

**Table 1:** Baseline characteristics, intraoperative variables, and postoperative outcomes of the cardiac-surgery cohort (n = 553), stratified by AKI status.

Variable	Total (n = 553)	AKI (n = 174)	No AKI (n = 379)	*p*-Value
Age, years (median [IQR])	69 [60–77]	77 [63–81]	68 [59–73]	<0.001
Male sex, n (%)	338 (61.1%)	102 (58.6%)	236 (62.3%)	0.469
BMI, kg/m^2^ (median [IQR])	27.3 [24.2–30.7]	26.3 [24.2–30.2]	27.4 [24.2–30.9]	0.587
Hypertension, n (%)	291 (52.6%)	89 (51.1%)	202 (53.3%)	0.705
Diabetes mellitus, n (%)	176 (31.8%)	52 (29.9%)	124 (32.7%)	0.572
COPD, n (%)	68 (12.3%)	17 (9.8%)	51 (13.5%)	0.277
Preop haemoglobin, g/dL	12.0 [10.4–13.6]	11.4 [10.0–12.8]	12.3 [10.6–14.0]	<0.001
ASA ≥ IV, n (%) ^a^	107 (19.3%)	31 (17.8%)	76 (20.1%)	0.615
**PROCEDURE TYPE**				
Isolated CABG, n (%)	218 (39.4%)	92 (52.9%)	126 (33.2%)	<0.001
Isolated valve, n (%)	173 (31.3%)	40 (23.0%)	133 (35.1%)	0.006
Combined CABG + Valve, n (%)	99 (17.9%)	27 (15.5%)	72 (19.0%)	0.383
Aortic procedure, n (%)	46 (8.3%)	14 (8.0%)	32 (8.4%)	1.000
Other cardiac surgery, n (%)	17 (3.1%)	1 (0.6%)	16 (4.2%)	0.018
**INTRAOPERATIVE**				
Aortic cross-clamp time, min ^b^	67 [45–99]	56 [39–93]	70 [47–100]	0.043
Intraoperative RBC units	2 [0–3]	2 [1–4]	1.5 [0–3]	0.017
Intraoperative FFP units	1 [0–2]	1 [0–3]	0 [0–2]	0.103
Any intraop inotrope, n (%)	235 (42.5%)	56 (32.2%)	179 (47.2%)	0.001
**POSTOPERATIVE**				
Mechanical ventilation, h	19 [11–65]	72 [27–82]	17 [10–22]	<0.001
Postop creatinine, mg/dL	1.3 [0.9–2.0]	1.9 [1.2–3.4]	1.2 [0.9–1.7]	<0.001
Postop eGFR, mL/min/1.73 m^2^	55.7 [25.2–84.4]	27.0 [15.0–53.8]	62.0 [33.0–85.9]	<0.001
**OUTCOME**				
In-hospital mortality, n (%)	92 (16.6%)	38 (21.8%)	54 (14.2%)	0.035

*Continuous variables presented as median [IQR]; categorical presented as n (%). p-values from Mann–Whitney U test for continuous and Chi-square or Fisher’s exact test for categorical variables. AKI defined by KDIGO criteria. ^a^ ASA classification recorded in 396/553 (71.6%). ^b^ Aortic cross-clamp time available in 482/553 (87.2%).*

**Table 2:** Additional preoperative laboratory values and postoperative clinical outcomes (cardiac-surgery cohort, n = 553).

Variable	Total (n = 553)	AKI (n = 174)	No AKI (n = 379)	*p*-Value
Haematocrit, %	38.5 [34.3–42.4]	37.2 [32.8–41.6]	38.8 [34.4–42.8]	0.131
WBC, ×10^9^/L	7.6 [6.1–9.4]	8.0 [6.6–9.8]	7.5 [6.0–9.3]	0.127
Neutrophils, ×10^9^/L	4.4 [3.4–5.9]	5.0 [3.6–7.5]	4.4 [3.4–5.6]	0.049
BUN, mg/dL	22 [17–33]	25 [19–37]	21 [16–32]	0.006
Preop creatinine, mg/dL	1.0 [0.8–1.4]	1.1 [0.8–1.4]	1.0 [0.8–1.4]	0.994
Preop eGFR, mL/min/1.73 m^2^	60.7 [26.0–88.0]	38.1 [15.0–79.7]	63.7 [32.0–88.8]	<0.001
Preop LVEF, %	55 [45–60]	55 [45–60]	55 [46–60]	0.605
Postop haemoglobin, g/dL	9.1 [8.5–9.8]	9.0 [8.4–9.8]	9.1 [8.5–9.8]	0.776
Postop RBC units	1 [0–2]	2 [1–3]	1 [0–2]	0.001
Surgical re-exploration, n (%)	50 (12.8%)	13 (10.9%)	37 (13.6%)	0.572
Delirium, n (%)	15 (3.8%)	0 (0.0%)	15 (5.5%)	0.007
ICU readmission, n (%)	70 (12.7%)	16 (9.2%)	54 (14.2%)	0.128
Postop inotrope, n (%)	226 (40.9%)	54 (31.0%)	172 (45.4%)	0.002

*Continuous variables presented as medians [IQR], categorical as n (%). Comparison: Mann–Whitney U or Chi-square/Fisher’s exact test as appropriate. For variables with partial documentation (surgical re-exploration, delirium, ICU readmission, and postoperative inotrope), denominators reflect patients with the variable recorded; see Section 3.3 (Missing Data) and Supplementary Methods for the missingness pattern.*

**Table 3:** Sequential multivariable logistic regression for acute kidney injury (cardiac-surgery cohort, n = 553). Models reported: A (baseline only), B (+procedure type), and C (+intraoperative variables).

Variable (AKI Outcome)	Univariable OR (95% CI), *p*	Model A: Baseline	Model B: +Procedure	Model C: Full
Haemoglobin (per 1 g/dL)	0.86 (0.79–0.94), <0.001	0.91 (0.81–1.03), 0.124	0.91 (0.80–1.03), 0.122	0.89 (0.73–1.08), 0.238
eGFR (per 1 mL/min/1.73 m^2^)	0.99 (0.98–0.99), 0.001	0.99 (0.98–1.00), 0.023	0.99 (0.98–1.00), 0.034	0.99 (0.98–1.00), 0.163
Age (per 1 year)	1.04 (1.03–1.06), <0.001	0.99 (0.96–1.01), 0.205	0.99 (0.96–1.01), 0.210	0.98 (0.95–1.02), 0.381
Intraop RBC units (per unit)	1.09 (1.02–1.16), 0.015	—	—	1.12 (1.00–1.25), 0.046
Intraop inotrope (any)	0.51 (0.35–0.75), 0.001	—	—	0.32 (0.13–0.81), 0.016
Procedure type (vs. Isolated CABG)				
Isolated valve	0.41 (0.26–0.64), <0.001	—	0.84 (0.43–1.62), 0.597	1.13 (0.39–3.31), 0.828
CABG + Valve	0.51 (0.31–0.86), 0.012	—	0.60 (0.28–1.28), 0.185	1.00 (0.34–2.95), 0.995
Aortic	0.60 (0.30–1.19), 0.142	—	0.76 (0.30–1.91), 0.555	0.96 (0.18–5.10), 0.968
Cross-clamp time (per 10 min)	0.99 (0.94–1.03), 0.565	—	—	1.06 (0.94–1.20), 0.368
**Model fit**				
AUROC	—	0.640	0.652	0.672
Hosmer–Lemeshow *p*	—	0.50	—	0.478
Sample size (n)	—	392	392	235

*OR = odds ratio; CI = confidence interval; CABG = coronary artery bypass grafting; RBC = red blood cell. Sample sizes differ across models reflecting variable-specific missingness; complete-case analysis for each specification. The haemoglobin–AKI association attenuates and loses statistical significance after adjustment, while intraoperative RBC transfusion remains independently associated in the fully adjusted Model C.*

**Table 4:** Sequential multivariable logistic regression for in-hospital mortality (cardiac-surgery cohort, n = 553). Models reported: Crude, A (+baseline), B (+procedure type), and C (+intraoperative variables).

Variable (Mortality Outcome)	Crude OR (95% CI), *p*	Model A: Baseline	Model B: +Procedure	Model C: Full
AKI (binary)	1.74 (1.09–2.79), 0.021	1.98 (1.03–3.82), 0.041	2.21 (1.10–4.46), 0.027	7.14 (1.45–35.13), 0.016
Age (per 1 year)	—	1.05 (1.02–1.08), 0.004	1.06 (1.03–1.09), 0.001	1.16 (1.04–1.29), 0.006
Haemoglobin (per 1 g/dL)	—	0.91 (0.79–1.04), 0.163	0.92 (0.80–1.05), 0.235	0.95 (0.71–1.28), 0.759
eGFR (per 1 mL/min/1.73 m^2^)	—	0.99 (0.98–1.00), 0.087	0.99 (0.98–1.00), 0.052	0.98 (0.96–1.00), 0.088
Cross-clamp time (per 10 min)	—	—	—	1.30 (1.06–1.59), 0.010
Intraop RBC units (per unit)	—	—	—	1.48 (1.20–1.83), <0.001
AUROC/HL *p*/n	—	0.704/0.62/392	0.748/0.45/392	0.925/0.559/235

*OR = odds ratio; CI = confidence interval; AUROC = area under ROC curve. The AKI–mortality association became substantially stronger after full adjustment (crude OR 1.74 → fully-adjusted Model C OR 7.14), supporting AKI as a robust independent predictor of in-hospital mortality. The high AUROC of Model C (0.925) should be interpreted cautiously: it was estimated in a smaller complete-case subset (n = 235) and may be subject to optimism and selection effects; the model was not internally validated for clinical prediction.*

**Table 5:** Non-linearity testing for continuous predictors and calibration of fully adjusted models.

Variable	Linear AIC	Quadratic AIC	LR Chi^2^ (*p*-Value)
Haemoglobin (AKI outcome)	384.8	386.8	0.04 (*p* = 0.835)
eGFR (AKI outcome)	384.8	386.4	0.36 (*p* = 0.547)
Creatinine (AKI outcome)	625.7	614.6	13.13 (*p* < 0.001)
**Calibration of fully adjusted models**			
AKI Model C	AUROC = 0.672	HL χ^2^ = 7.56	*p* = 0.478 (good)
Mortality Model C	AUROC = 0.925	HL χ^2^ = 6.79	*p* = 0.559 (good)

*LR = likelihood-ratio test (1 df) comparing linear vs. quadratic specifications for each continuous predictor. AIC = Akaike Information Criterion. AUROC = area under ROC curve. HL = Hosmer–Lemeshow goodness-of-fit test (8 df). Both fully adjusted models show acceptable calibration (HL p > 0.05). Creatinine shows non-linearity (threshold pattern); CKD-EPI eGFR—which incorporates a non-linear age-creatinine transformation—is therefore preferred as the principal renal-function variable.*

**Table 6:** Categorical creatinine analysis and effect sizes in clinically interpretable units.

Creatinine Category (mg/dL)	n (Cardiac)	AKI Events, n (%)	Adjusted OR (95% CI)
<1.0 (reference)	180	56 (31.1%)	1.00 (ref)
1.0–1.3	157	44 (28.0%)	0.75 (0.41–1.36)
1.3–2.0	131	40 (30.5%)	0.62 (0.31–1.27)
>2.0	82	30 (36.6%)	0.92 (0.39–2.16)
**Effect sizes in clinical units**			
Hb per 1 g/dL decrease (univariable)	—	—	1.16 (1.07–1.26)
eGFR per 10 mL/min decrease (univariable)	—	—	1.15 (1.06–1.24)

**Table 7:** Descriptive characteristics of the cardiac-surgery cohort and the carotid endarterectomy subgroup (reported for transparency only; the two groups are not directly comparable—see Limitations).

Variable	Cardiac Surgery (n = 553)	Carotid Endarterectomy (n = 150)
Age, years	69 [60–77]	64 [58–70]
Male sex, n (%)	338 (61.1%)	107 (71.3%)
Preop haemoglobin, g/dL	12.0 [10.4–13.6]	13.5 [12.3–14.7]
Preop eGFR, mL/min/1.73 m^2^	60.7 [26.0–88.0]	81.4 [65.1–92.9]
Preop creatinine, mg/dL	1.0 [0.8–1.4]	0.9 [0.8–1.1]
Acute kidney injury, n (%)	174 (31.5%)	21 (14.0%)
In-hospital mortality, n (%)	92 (16.6%)	0 (0.0%)

*Reported here for transparency only. The two groups differ substantially in clinical complexity, baseline risk, and operative exposure (cardiopulmonary bypass vs. no bypass); they are not directly comparable, and no causal inference is drawn from this subgroup. See Limitations. Categorical Cr analysis adjusted for age, sex, and haemoglobin. The threshold pattern (highest AKI rate at >2 mg/dL) supports the use of CKD-EPI eGFR (which includes non-linear age-creatinine transformation) as the principal renal-function variable.*

## Data Availability

The data presented in this study are available on reasonable request from the corresponding author. The data are not publicly available due to privacy and ethical restrictions related to patient confidentiality, in accordance with the institutional ethics committee approval.

## Supplementary Materials

The following supporting information can be downloaded at: https://www.mdpi.com/article/10.3390/jcm15124498/s1, Figure S1: Directed acyclic graph (DAG) representing the assumed causal structure underlying the analysis.
