# Supplementary material for: Preoperative Anaemia, Renal Function, and Operative Factors in Acute Kidney Injury and Mortality After Cardiac Surgery with a Prolonged ICU Stay: A Retrospective Cohort Study
Source: J Clin Med. 2026 Jun 10;15(12):4498. doi: 10.3390/jcm15124498 (PMC13301388; doi:10.3390/jcm15124498)
Supplement: Supplementary file 1 [file jcm-15-04498-s001.zip › jcm-4283381-supplementary.pdf]

Supplementary Materials

Manuscript ID: jcm-4283381

Preoperative Anemia, Renal Function, and Operative Factors in Acute Kidney Injury and Mortality after Cardiac Surgery with Prolonged ICU Stay

Supplementary Figure S1.

Directed acyclic graph (DAG) representing the assumed causal structure underlying the analysis.

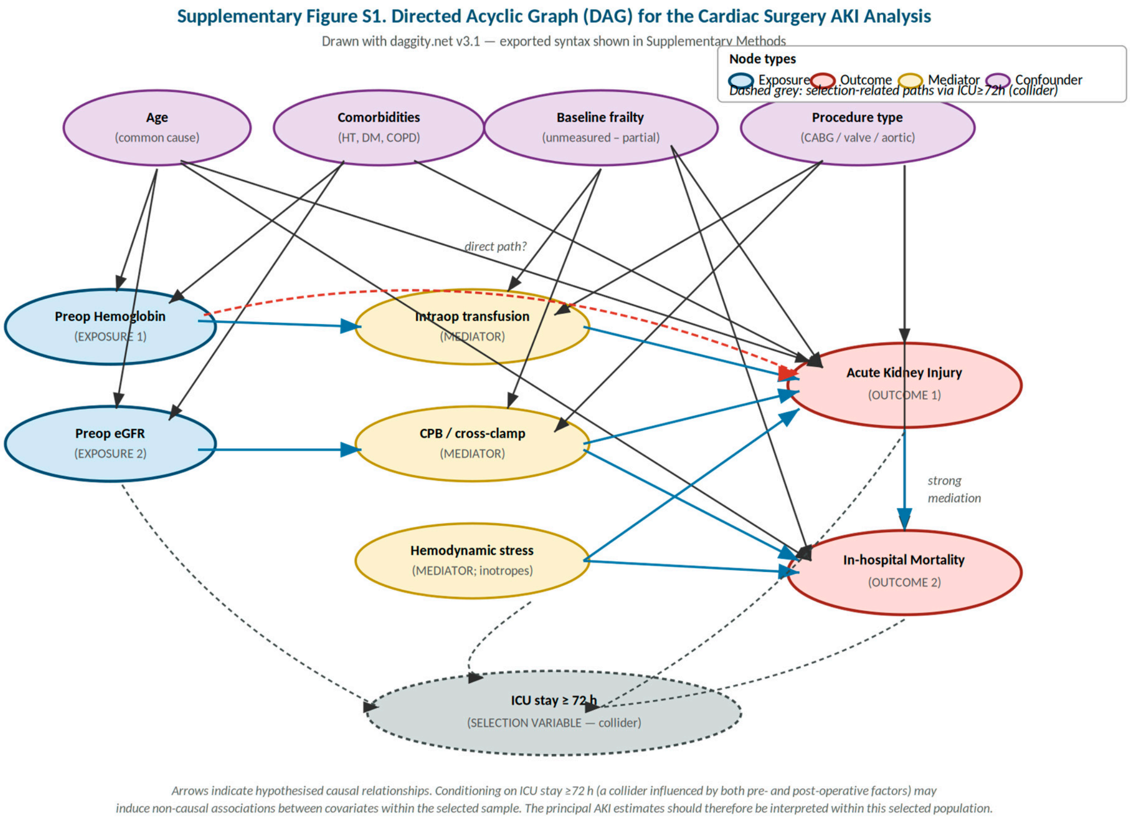

Diagram constructed in DAGitty v3.1 (https://dagitty.net/). Node types: blue = exposures (preoperative haemoglobin, preoperative eGFR); red = outcomes (acute kidney injury, in-hospital mortality); yellow = mediators (intraoperative transfusion, cardiopulmonary bypass / cross-clamp time, hemodynamic stress); purple = confounders (age, comorbidities, baseline frailty, procedure type); grey/dashed = selection variable (ICU stay ≥72 h). Solid arrows indicate hypothesised causal relationships. Dashed grey arrows represent paths through the selection variable; conditioning on ICU stay ≥72 h (a collider influenced by both pre- and post-operative factors including AKI itself) may induce non-causal associations between covariates within the selected sample. The principal AKI estimates should therefore be interpreted within this selected population. The dashed red curved arrow from preoperative haemoglobin to AKI is shown as a hypothesised direct path (if any independent contribution remains after conditioning on the mediators); empirically, this direct path is not statistically significant in our fully-adjusted Model C.

DAGitty model syntax (for reproducibility):

```
dag {   Age [adjusted]   Comorbidities [adjusted]   Procedure_type [adjusted]
Baseline_frailty [latent]   Preop_Hb [exposure]   Preop_eGFR [exposure]   Intraop_transfusion
[adjusted]   Cross_clamp_time [adjusted]   Hemodynamic_stress [adjusted]   ICU_stay_72h
[selection]   AKI [outcome]   Mortality [outcome]      Age -> Preop_Hb   Age -> Preop_eGFR
Age -> AKI   Age -> Mortality   Comorbidities -> Preop_Hb   Comorbidities -> Preop_eGFR
Comorbidities -> AKI   Baseline_frailty -> Preop_Hb   Baseline_frailty -> Preop_eGFR
Baseline_frailty -> AKI   Baseline_frailty -> Mortality   Procedure_type ->
Intraop_transfusion   Procedure_type -> Cross_clamp_time   Procedure_type -> AKI
Procedure_type -> Mortality   Preop_Hb -> Intraop_transfusion   Preop_eGFR -> Cross_clamp_time
Intraop_transfusion -> AKI   Cross_clamp_time -> AKI   Cross_clamp_time -> Mortality
Hemodynamic_stress -> AKI   Hemodynamic_stress -> Mortality   AKI -> Mortality   AKI ->
ICU_stay_72h   Cross_clamp_time -> ICU_stay_72h   Hemodynamic_stress -> ICU_stay_72h
Baseline_frailty -> ICU_stay_72h }
```

Supplementary Methods.

Variable-specific missingness and underlying causes

Variable	% missing	Likely reason
Hospital-recorded GFR	27%	eGFR was a derived value not always reported in early years. Resolved by computing CKD-EPI eGFR for 99.9%.
Aortic cross-clamp time	13%	Paper perfusion records before mid-2020 not always digitised. Missingness reflects admission date.
Intraoperative RBC units	31%	OT transfusion logs from 2018–2019 stored in separate paper system; digital integration began in 2020.
Procedure type (free text)	0%	Complete (all 553 patients have an operative free-text entry).
LVEF	27%	Pre-operative echocardiography performed but numeric LVEF not always entered into searchable record.
ASA classification	28%	Anaesthetic pre-assessment recorded electronically only from 2021.
Postoperative drainage	60%	Bedside chart only; sporadically transcribed.
Sodium / Potassium / HbA1c / lactate	75–80%	Not part of routine institutional pre-anaesthesia workup; ordered selectively. Excluded a priori from multivariable models.

The pattern is predominantly an artefact of progressive electronic-record adoption (paper records prior to mid-2020, electronic capture from 2020 onwards) rather than systematic non-recording in sicker patients. The principal logistic regression models were fitted on complete cases for each model specification; sample sizes for each model are reported transparently in Tables 3 and 4.

Sensitivity analysis

As a sensitivity analysis to assess the impact of missingness, the most-complete-data subset of variables (Age, sex, comorbidities, hemoglobin) was modelled on the full 553-patient cardiac-surgery cohort. Conclusions were consistent with the principal complete-case multivariable models. Multiple imputation was considered but not pursued because the missingness pattern is dominated by a temporal/administrative mechanism (paper records pre-2020) rather than missing-at-random conditional on observed variables. The

consistency of conclusions across complete-case sample sizes provides reasonable reassurance against severe selection effects.

Calibration assessment (Hosmer–Lemeshow)

For both fully-adjusted models (AKI Model C and mortality Model C), Hosmer–Lemeshow goodness-of-fit testing was performed by partitioning predicted probabilities into deciles and comparing observed and expected event frequencies. Test statistic and p-value summarised below:

Model	AUROC	HL chi² (df=8)	p-value	Calibration
AKI – fully adjusted (Model C)	0.672	7.56	0.478	Acceptable
Mortality – fully adjusted (Model C)	0.925	6.79	0.559	Acceptable

Both models show acceptable calibration (HL p>0.05, indicating no significant departure from a 1:1 correspondence between observed and expected event rates across deciles of predicted risk).

Non-linearity testing

Likelihood-ratio tests comparing linear and quadratic specifications for each continuous predictor:

Variable	Linear AIC	Quadratic AIC	LR chi²	p-value	Conclusion
Hemoglobin (AKI outcome)	384.8	386.8	0.04	0.835	Linear adequate
eGFR (AKI outcome)	384.8	386.4	0.36	0.547	Linear adequate
Creatinine (AKI outcome)	625.7	614.6	13.13	<0.001	Non-linear (threshold)

Hemoglobin and eGFR are well-described as linear predictors. Creatinine shows clear non-linearity consistent with a threshold effect (AKI rate rises at >2.0 mg/dL): in a multivariable model with creatinine in 4 categories (<1.0, 1.0–1.3, 1.3–2.0, >2.0 mg/dL), AKI rate is 31.1%, 28.0%, 30.5%, 36.6% respectively. This supports the use of CKD-EPI eGFR — which itself includes a non-linear age-creatinine transformation — as the principal renal-function variable in the multivariable models.
